# Patient-level pooled analysis of adjudicated gastrointestinal outcomes in celecoxib clinical trials: meta-analysis of 51,000 patients enrolled in 52 randomized trials

**DOI:** 10.1186/ar4134

**Published:** 2013-01-08

**Authors:** Andrew Moore, Geoffrey Makinson, Chunming Li

**Affiliations:** 1 Pain Research, Nuffield Division of Anaesthetics, Nuffield Department of Clinical Neuroscience, University of Oxford, The Churchill, Oxford, OX3 7LE, UK; 2 Medical Affairs, Primary Care Unit, Pfizer Inc, 235 E 42nd Street, New York, NY 10017, USA; 3 Statistics, Primary Care Unit, Pfizer Inc, 235 E 42nd Street, New York, NY 10017, USA

## Abstract

**Introduction:**

Although the safety of celecoxib has been investigated, limited data are available on complications affecting the entire (upper and lower) gastrointestinal (GI) tract, with no patient-level pooled analyses of upper and lower GI outcomes available. We therefore evaluated the upper and lower GI safety of celecoxib by using patient-level data from randomized controlled trials (RCTs).

**Methods:**

This patient-level pooled analysis included 52 prospective, randomized, double-blind parallel-group studies from the Celecoxib Clinical Database. Each study had a planned duration of continuous treatment with celecoxib or a nonselective nonsteroidal antiinflammatory drug (nsNSAID), rofecoxib, or the placebo comparator arm for at least 4 weeks. All studies with final reports completed by 1 October 2007 were included. The primary end point was the combined incidence of clinically significant upper and lower GI events (CSULGIEs). An independent blinded committee reviewed and adjudicated all end points by using predefined criteria and all available reported adverse events, laboratory data, and case narratives. All doses of celecoxib and all doses of all nsNSAIDs were pooled for analysis.

**Results:**

The pooled analysis involved 51,048 patients; 28,614 were randomized to celecoxib; 15,278 to nsNSAIDs (including 3,248 patients taking naproxen, 2,640 taking ibuprofen, 8,066 taking diclofenac, 1,234 taking loxoprofen, and 90 taking ketoprofen); 5,827 to placebo and 1,329 to rofecoxib. The mean age was 60 years, and 65% were women. Data on 1,042 patients with potential GI events were reviewed for end-points adjudication; the adjudication committee confirmed 89 patients with CSULGIEs. The majority were in the celecoxib and nsNSAID groups (with raw incidence proportions of 37 (0.1%) and 40 (0.3%), respectively). The incidence rates were 0.3, 0.9 and 0.3 per 100 patient-years in the celecoxib, nsNSAID, and placebo groups, respectively. The time to incidence of CSULGIEs was significantly longer with celecoxib than with nsNSAIDs (*P *= 0.0004).

**Conclusions:**

When compared with nsNSAIDs, celecoxib is associated with a significantly lower risk of all clinically significant GI events throughout the entire GI tract. This pooled analysis of 52 RCTs significantly advances the understanding of the upper and lower GI safety profile of celecoxib and its potential benefits to patients.

## Introduction

Patients seeking effective pain relief from chronic and acute painful conditions such as osteoarthritis (OA), rheumatoid arthritis (RA), postsurgical pain, and dysmenorrhea may be prescribed nonselective (ns) nonsteroidal antiinflammatory drugs (NSAIDs) (for example, naproxen, diclofenac, and ibuprofen) or cyclooxygenase (COX)-2 selective NSAIDs (for example, celecoxib). The benefits and risks of each medication vary according to both the clinical setting and individual patient characteristics. Some patients respond well to NSAID use, whereas others receive little benefit [1]. However, all patients using NSAIDs run the risk of associated adverse events (AEs).

The AEs associated with NSAID use include upper and lower gastrointestinal (GI) adverse outcomes [2-6] and cardiovascular events [7-10]. Assessment of both upper- and lower-GI complications is important, as a number of studies have shown that the use of NSAIDs increases the risk of lower-GI AEs [5,11,12]. The novel end point of clinically significant upper-and lower-GI events (CSULGIEs) extends the traditional assessment of upper-GI complications (perforations, obstructions and bleeding in the esophagus, stomach, and duodenum) by also including events (perforation, bleeding, and clinically significant anemia) from the lower-GI tract (small/large bowel) [2].

Unlike nsNSAIDs that inhibit both the constitutive and inducible forms of the COX enzyme (COX-1 and COX-2), celecoxib is selective for the COX-2 enzyme. Inhibition of the COX-1 enzyme is responsible for the associated GI toxicity of nsNSAIDs [13], and the selective COX-2 inhibitory activity of celecoxib is believed to be associated with a lower risk of GI AEs than are the nsNSAIDs [14].

The GI safety of celecoxib has been investigated in observational studies, randomized controlled trials (RCTs), and meta-analyses of RCTs. An observational study found a lower short-term risk of upper-GI toxicity for celecoxib than for nsNSAIDs, as measured in hospitalization rates due to upper-GI bleeds [15]. Two large RCTs, Celecoxib Long-term Arthritis Safety Study (CLASS) and SUccessive Celecoxib Efficacy and Safety Study (SUCCESS), compared the GI safety of celecoxib with that of nsNSAIDs by using the upper-GI ulcer-complications measurement. CLASS found celecoxib to be no different from comparator nsNSAIDs in the incidence of upper-GI ulcer complications [16], whereas SUCCESS showed superior GI safety of celecoxib over nsNSAIDs [17]. A third large RCT, Celecoxib vs Omeprazole and Diclofenac in Patients with Osteoarthritis and Rheumatoid Arthritis (CONDOR), compared the total (upper and lower) GI safety of celecoxib with that of the nsNSAID diclofenac in patients with OA and RA at increased gastrointestinal risk. This was the first clinical trial to show that the risk of clinical outcomes throughout the GI tract was significantly reduced in patients treated with celecoxib compared with diclofenac-treated patients [18].

Meta-analyses of RCTs, comparing celecoxib with nsNSAIDs for upper-GI events, also concluded that celecoxib is superior to comparator nsNSAIDs for GI safety [19-21]. The review by Deeks *et al. *(2002) was limited to nine trials and 15,187 patients; the review by Moore *et al. *(2005) was a more comprehensive analysis of 31 RCTs and 39,605 patients based on Pfizer study reports, but did not assess ulcer complications; and the Rostom *et al. *(2007) review was not limited to celecoxib trials. None of these meta-analyses, however, included patient-level data with independent adjudication.

Although the safety of celecoxib has previously been investigated in observational studies, RCTs, and meta-analyses, limited data are available on complications affecting the entire (upper and lower) GI tract. To date, no patient-level comprehensive meta-analysis or pooled analysis of both upper- and lower-GI outcomes with COX-2 selective NSAIDs has been published. The present pooled analysis evaluating the upper- and lower-GI safety of celecoxib in RCTs fills this evidence gap.

## Methods

### Clinical studies

All clinical studies from the Pfizer Celecoxib Clinical Database meeting the following criteria were identified and included in the pooled analysis: (a) randomized, double-blind studies with a parallel-group design; (b) one treatment arm was celecoxib with placebo or nsNSAID comparator; (c) planned duration of continuous treatment ≥ 4 weeks; and (d) final study report completed by 1 October 2007. Open-label, crossover trials, and all studies of healthy volunteers were excluded from the pooled analysis.

At the time of the analysis, Pfizer was concurrently conducting large clinical research programs (with up to 30,000 subjects) to assess GI and/or cardiovascular events associated with the use of celecoxib and commonly used NSAIDs. However, because CONDOR (NCT00141102) [18], Gastrointestinal Randomized Event And Safety Open-Label NSAID Study (GI-REASONS; NCT00373685) and the Prospective Randomized Evaluation of Celecoxib Integrated Safety Vs Ibuprofen or Naproxen (PRECISION; NCT00346216) trial were ongoing at the time of analysis, data from these studies were not available for inclusion in the pooled analysis presented here.

### Outcome measures

The primary end point was the cumulative incidence of CSULGIEs (including adjudicated perforations, obstructions, and clinically significant bleeds (defined as overt bleeds or a decrease in hemoglobin of ≥ 2 g/dl and/or hematocrit ≥ 10% of GI origin)). The definition of CSULGIE used by the independent adjudication committee was that proposed by Chan and colleagues (Table 1) [2].

**Table 1:** Clinically significant upper- and lower-GI events (CSULGIEs) composite end point

With lesion	Without lesion
GD hemorrhage	Acute hemorrhage of unknown origin, including presumed small bowel hemorrhage

Gastric-outlet obstruction	Clinically significant anemia of presumed occult GI origin, including possible small bowel blood loss

GD, small-bowel, or large-bowel perforation	

Large-bowel hemorrhage	

Small-bowel hemorrhage	

Small-bowel obstruction	

Clinically significant anemia of defined GI origin	

Symptomatic ulcers	

GD, gastroduodenal; GI, gastrointestinal.

Secondary end points included the incidence of CSULGIEs or symptomatic ulcers in patients with or without a hemoglobin decrease ≥ 2 g/dl of GI-related or potential GI cause.

## GI end-point adjudication committee

An independent blinded expert committee adjudicated all end points by using predefined GI-complications criteria (gastroduodenal, small bowel, or large bowel perforation; gastric outlet obstruction; gastroduodenal hemorrhage; large bowel hemorrhage; small bowel hemorrhage; clinically significant anemia of defined GI origin; acute GI hemorrhage of unknown origin, including presumed small bowel hemorrhage; and clinically significant anemia of presumed occult GI origin, including possible small bowel blood loss) as well as available reported AEs, laboratory data, vital signs, and case narratives, where available.

The adjudication committee consisted of two external, independent gastroenterologists and one external independent rheumatologist. The two gastroenterologists acted as adjudicators, and the rheumatologist acted as a facilitator only for cases in which agreement was not initially reached between the adjudicators. The adjudicators advised on the proper definition of potential and suspected outcomes; they provided guidance on the selection of events to be adjudicated, guidance regarding evaluation, and collection of information necessary to adjudicate suspected GI outcomes and guidance on the screening methods to be applied to potential events before adjudication. Additionally, the adjudicators validated the screening method of the potential events, reviewed, in a blinded fashion, and where necessary, rereviewed all suspected GI outcomes by using the clinical information supplied in supporting documentation, and provided final classification of the primary and secondary events as an end point or not an end point. Adjudication of CSULGIEs could be made satisfactorily only when clinical information in the form of a narrative was available. The facilitator coordinated consensus between adjudicators in cases of discordance, but did not adjudicate any end points.

### Statistical analysis

Subjects were included in the safety analysis if they were randomized and treated with one or more doses of study medication. All doses of celecoxib (from < 200 to 800 mg total daily dose), and all doses of all nsNSAIDs were pooled for analysis. Tabulations and descriptive statistics were used to summarize end points of interest; 95% confidence intervals are presented, if applicable, and all hypothesis testing was two-sided, with a significance level of 5%.

Statistical procedure LIFETEST in SAS software (Version 8 of the SAS System) was used to compute and plot the estimate of the distribution of the survival time (that is, the Kaplan-Meier curve). A stratified log-rank test was used to compare treatments, adjusting for studies.

In addition to the raw events, exposure-adjusted incidence rates (number of patients with events per 100 years of exposure) were calculated to account for potential differences between groups in duration of exposure to treatment. The incidence rates were calculated by dividing the number of adjudicated GI events by the total number of years that patients were treated in the study.

## Results

### Clinical studies included

Fifty-two studies were identified as meeting the selection criteria (see Additional file 1). Of these, 37 studies were in OA/RA, involving 41,638 (82%) patients; five studies in chronic low-back pain involved 3,635 (7%) patients, four in ankylosing spondylitis involved 1,644 (3%) patients, three in Alzheimer disease involved 487 (1%) patients, and three in oncology involved 3,644 (7%) patients. The mean age was about 60 years, and 65% were women.

**Figure 1:**
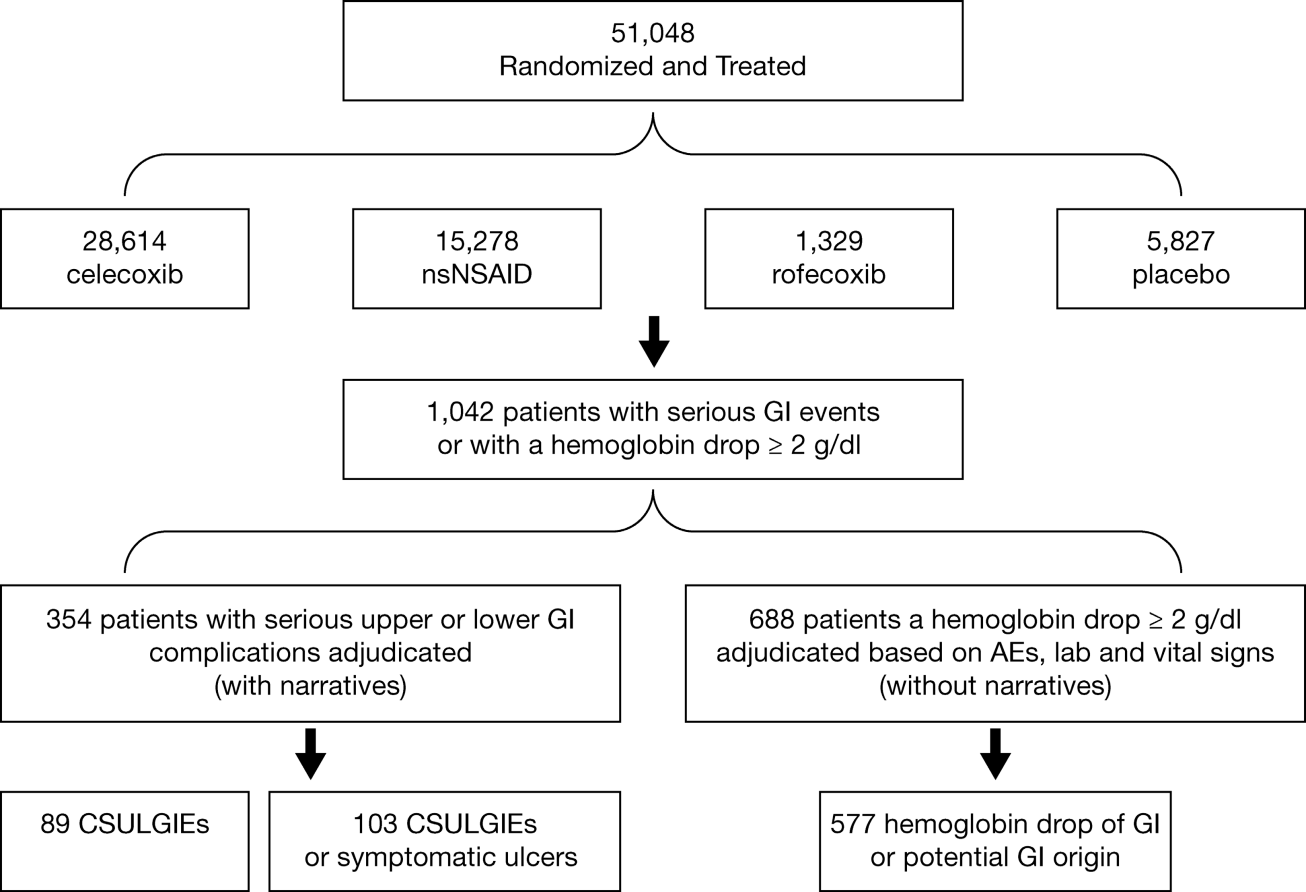
**Incidence of CSULGIEs**. AEs, adverse events; CSULGIEs, clinically significant upper and lower GI events; GI, gastrointestinal; nsNSAID, nonselective nonsteroidal antiinflammatory drug.

As shown in Table 2 and Figure 1, 51,048 patients were included in this pooled analysis; 28,614 patients were randomized to celecoxib (875 patients taking < 200 mg, 12,835 taking 200 mg, 9,616 taking 400 mg, and 5,288 taking 800 mg total daily dose); 15,278 to nsNSAIDs (including 3,248 patients taking naproxen, 2,640 taking ibuprofen, 8,066 taking diclofenac, 1,234 taking loxoprofen, and 90 taking ketoprofen); 5,827 to placebo, and 1,329 to rofecoxib. The majority (60%) of the trials were ≤ 12 weeks in duration. The total patient-years of exposure were 20,808: 12,276 years in the celecoxib group, 4,622 years in the nsNSAID group, 3,756 years in the placebo group, and 155 years in the rofecoxib group.

**Table 2:** Baseline patient characteristics

	Celecoxib (*n *= 28,614)	nsNSAID (*n *= 15,278)	Placebo (*n *= 5,827)	Rofecoxib (*n *= 1,329)	Total (*n *= 51,048)
Age, years					

Mean, SD	60.1 (13)	59.3 (13)	57.2 (14)	70.6 (9)	59.8 (13)

≥ 65 years, *n *(%)	11,404 (40)	5,667 (37)	1,901 (33)	1,115 (84)	20,087 (39)

≥ 75 years, *n *(%)	3,421 (12)	1,656 (11)	513 (9)	460 (35)	6,050 (12)

Female, *n *(%)	18,819 (66)	10,478 (69)	3,251 (56)	860 (65)	33,408 (65)

Indication					

OA/RA, *n *(%)	23,324 (82)	13,911 (91)	3,074 (53)	1,329 (100)	41,638 (82)

Low-back pain, *n *(%)	1,743 (6)	850 (6)	1,042 (18)	0 (0)	3,635 (7)

Ankylosing spondylitis, *n *(%)	895 (3)	517 (3)	232 (4)	0 (0)	1,644 (3)

Alzheimer disease, *n *(%)	329 (1)	0 (0)	158 (3)	0 (0)	487 (1)

Oncology, *n *(%)	2,323 (8)	0 (0)	1,321 (23)	0 (0)	3,644 (7)

Duration, *n *(%)					

≤ 12 weeks	15,597 (55)	8,322 (55)	3,871 (66)	1,245 (94)	29,035 (57)

> 12 and ≤ 26 weeks	7,878 (28)	4,271 (28)	675 (12)	84 (6)	12,908 (25)

> 26 and ≤ 52 weeks	2,307 (8)	1,958 (13)	90 (2)	0 (0)	4,355 (9)

≥ 52 weeks	2,832 (10)	727 (5)	1,191 (20)	0 (0)	4,750 (9)

Total patient-years of exposure	12,276.3	4,621.8	3,755.6	154.5	20,808.2

100 patient-years of exposure	122.8	46.2	37.6	1.6	208.1

100 Patient-years of exposure = total patient-years of exposure divided by 100. nsNSAID, nonselective nonsteroidal antiinflammatory drug; OA, osteoarthritis; RA, rheumatoid arthritis.

In total, 1,042 patients with either a reported serious GI event (354 patients) or with a hemoglobin decrease ≥ 2 g/dl (688 patients; Figure 1) were sent for adjudication (celecoxib *n *= 489 (2%); nsNSAIDs *n *= 456 (3.0%); placebo *n *= 92 (1.6%); rofecoxib *n *= 5 (0.4%)). Of the 354 serious GI cases with narratives, 203 (0.7%) were in the celecoxib group, 104 (0.7%) in the nsNSAID group, 46 (0.8%) in the placebo group, and 1 (0.1%) in the rofecoxib group. The 688 patients with a decrease in hemoglobin ≥ 2 g/dl without a narrative were adjudicated based on additional information, including AEs, laboratory values, and vital signs. The majority of these patients were from the celecoxib (286 (1.0%)) and nsNSAIDs groups (352 (2.3%)), followed by the placebo (46 (0.8%)) and rofecoxib groups (4 (0.3%)).

### Primary end point: incidence of CSULGIEs

**Figure 2:**
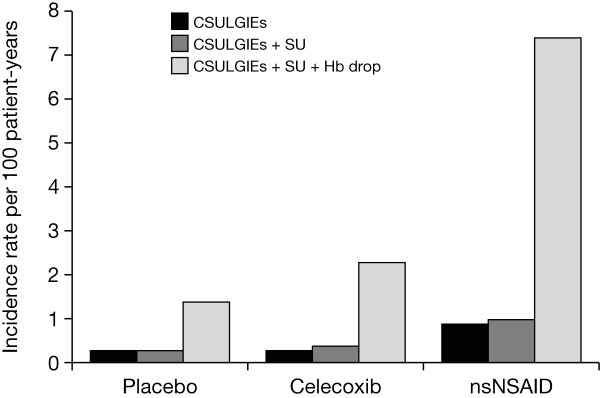
**Incidence rate (per 100 patient-years) of CSULGIEs**. Hb, hemoglobin; nsNSAID, nonselective nonsteroidal antiinflammatory drug; SU, symptomatic ulcer.

**Figure 3:**
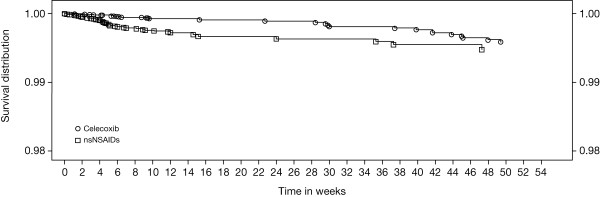
**Kaplan-Meier plot of time to incidence of CSULGIEs**. nsNSAID, nonselective nonsteroidal antiinflammatory drug.

Of the 354 patients with narratives, the adjudication committee confirmed 89 patients with CSULGIEs (Table 3). The majority of these patients were in the celecoxib and nsNSAID groups (37 (0.1%) and 40 (0.3%) patients, respectively). The remaining 12 patients were from the placebo groups. The incidence rates were 0.3, 0.9, and 0.3 per 100 patient-years in the celecoxib, nsNSAID, and placebo groups, respectively (Figure 2). The time to incidence of CSULGIEs was significantly longer with celecoxib than with nsNSAIDs (*P *= 0.0004) (Figure 3).

**Table 3:** GI end points

	Celecoxib (*n *= 28,614)	nsNSAID (*n *= 15,278)	Placebo (*n *= 5,827)	Rofecoxib (*n *= 1,329)	Total (*n *= 51,048)
CSULGIEs, *n *(%)	37 (0.1)	40 (0.3)	12 (0.2)	0 (0)	89 (0.2)

Incidence rate^a^	0.3	0.9	0.3	0	0.4

CSULGIEs or symptomatic ulcers, *n *(%)	47 (0.2)	44 (0.3)	12 (0.2)	0 (0)	103 (0.2)

Incidence rate^a^	0.4	1.0	0.3	0	0.5

CSULGIEs, symptomatic ulcers, or hemoglobin decrease, *n *(%)	281 (1.0)	343 (2.3)	52 (0.9)	4 (0.3)	680 (1.3)

Incidence rate^a^	2.3	7.4	1.4	2.6	3.3

^a^Incidence rate based on 100 patient-years of exposure to drug. CSULGIEs, clinically significant upper and lower GI events; nsNSAID, nonselective nonsteroidal antiinflammatory drug.

### Secondary end points

#### Incidence of CSULGIES or symptomatic ulcers

The adjudication committee confirmed a CSULGIE or symptomatic ulcer in 103 of 354 patients with narratives (47 (0.2%) patients in celecoxib, 44 (0.3%) in nsNSAID, and 12 (0.2%) in placebo groups) (Table 3). The incidence rates were 0.4, 1.0, and 0.3 per 100 patient-years in the celecoxib, nsNSAID, and placebo groups, respectively (Figure 2). The time to incidence of CSULGIEs was significantly longer with celecoxib than with nsNSAIDs (*P *= 0.0006) (see Additional file 2).

#### Incidence of CSULGIES, or symptomatic ulcers, or hemoglobin decrease ≥ 2 g/dl

Of the 688 patients who had a decrease in hemoglobin ≥ 2 g/dl, the adjudication committee confirmed 577 patients as having significant bleeds of GI-related or potential GI cause. Combining with those CSULGIEs and symptomatic ulcers, a total of 680 patients met this secondary end point (Table 3). The majority of these patients were in the celecoxib and nsNSAID groups (281 and 343 patients, respectively). The remaining 56 patients were from the placebo (52 patients) and rofecoxib (four patients) groups. The incidence rates were 2.3, 7.4, 1.4, and 2.6 per 100 patient-years in the celecoxib, nsNSAID, placebo, and rofecoxib groups, respectively (Figure 2). The time to incidence of CSULGIEs was significantly longer with celecoxib than with nsNSAID (*P *< 0.0001) (see Additional file 3).

## Discussion

The findings of this retrospective pooled analysis of 52 RCTs and more than 51,000 patients show that celecoxib is associated with a significantly lower risk of all clinically significant GI events throughout the entire GI tract when compared with nsNSAIDs, however defined. This is consistent with previously published meta-analyses [19-21] and observational studies [15,22].

A major strength of this patient-level pooled analysis was the inclusion of more than 51,000 patients with active disease (OA/RA, chronic low-back pain, ankylosing spondylitis, Alzheimer disease, or cancer), giving a robust sample size. It included all the methodologically sound randomized trials of celecoxib performed of at least 4 weeks' duration and concluded by October 2007. Further strengths included the independent evaluation of both upper- and lower-GI complications, together with the blinded adjudication of cases.

Caution should be exercised, however, because many of the clinical trials included in this analysis were not designed to study serious GI outcomes, and, at the time, detailed clinical information was not available on all suspected end points. In addition, the quality and the availability of the narratives varied (that is, some narratives were not available, and some were more fully descriptive of the serious AEs than were others). The members of the adjudication committee used their best clinical judgments based on available data for these adjudications. Furthermore, analysis of trials solely from the Pfizer Celecoxib Clinical Database might exclude relevant trials conducted elsewhere.

One additional issue is that the number of primary outcome events (patients experiencing CSULGIEs) was, at 89, below the limits of about 200 to eliminate random-chance effects suggested for adverse-event determinations [23]. For secondary outcomes, the number of events was > 200. The primary and secondary events were influenced in the same direction and to much the same extent.

Statistical heterogeneity between trials is not unexpected when the number of events is small, as in the case of rare adverse events [24]. The primary analysis method using a stratified log-rank test adjusting for studies was prespecified. Small numbers of events produce uncertainty over results [25], with subgroup analysis based on small numbers producing incorrect results; therefore, the advice is not to consider subgroup analyses in this circumstance [26]. For these reasons, together with the adjudication of CSULGIEs as a specific definition rather than by site of injury (upper or lower gastrointestinal tract), no subgroup analyses were attempted.

**Figure 4:**
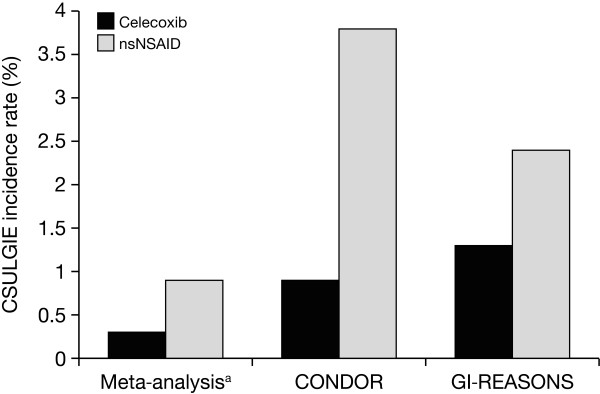
**CSULGIE incidence rates in the meta-analysis and subsequent large RCTs**. *Incidence rate based on 100 patient-years of exposure to drug. CONDOR, Celecoxib vs Omeprazole and Diclofenac in Patients with Osteoarthritis and Rheumatoid Arthritis study; GI-REASONS, Gastrointestinal Randomized Event And Safety Open-Label NSAID Study; nsNSAID, nonselective nonsteroidal antiinflammatory drug.

Systematic reviews generally should have a cut-off date beyond which additional information for new studies is not added. This is especially necessary when a detailed program of adjudication is required, as it was for this analysis of more than 1,000 patients. Medicines like celecoxib, with ongoing investigations into their benefits and safety, require the findings from meta-analyses to be continually compared with results from studies completed after the meta-analysis study inclusion cut-off date. This is particularly the case when the number of patients exposed or the duration of exposure would have added significantly to the total number available in the meta-analysis: here, 28,614 patients were exposed to celecoxib for 12,276 years. Several studies that were completed after the cut-off date are relevant, and show similar trends (Figure 4)

A large clinical research program of three studies (CONDOR, GI-REASONS, and PRECISION) to assess both the GI and/or cardiovascular safety of celecoxib and commonly used nsNSAIDs was ongoing at the cut-off date of this meta-analysis. The CONDOR and GI-REASONS studies have now been completed, whereas the PRECISION trial is still ongoing. The CONDOR trial, which compared the total GI safety of celecoxib with diclofenac plus omeprazole by using the novel composite GI end point of CSULGIEs, was completed in May 2009 [18]. OA and RA patients (*n *= 4,484) were randomized either to celecoxib or to diclofenac plus omeprazole for 6 months. The rate of CSULGIEs was significantly lower with celecoxib than with diclofenac plus omeprazole (0.9% versus 3.8%), and the majority of the events were related to anemia (decrease in hemoglobin ≥ 2 g/dl).

The GI-REASONS study, which was completed in November 2010, assessed the incidence of CSULGIEs in OA patients (*n *= 8,067) randomized to celecoxib or nsNSAIDs for 6 months [27]. The rate of CSULGIEs was significantly lower with celecoxib (1.3%) than with nsNSAIDs (2.4%), and the majority of events were also related to anemia (decrease in hemoglobin, ≥ 2 g/dl).

These two studies used the same end point as did our meta-analysis, used an adjudication committee, and reached essentially the same conclusion, that celecoxib was associated with a significantly lower rate of GI tract events than were nsNSAIDs. Other trials involving celecoxib have been reported since 2007, but none of a size or concentration on GI-tract outcomes that would allow comparison with results from our meta-analysis.

## Conclusions

When compared with nsNSAIDs, celecoxib is associated with a significantly lower risk of all clinically significant GI events throughout the entire GI tract. This pooled analysis of patient-level data from 52 RCTs significantly advances the understanding of the cumulative upper- and lower-GI safety profile of celecoxib and its potential benefits to patients. Future NSAID safety studies should consider analyzing the cumulative incidence of clinically significant upper- and lower-GI events.

## Abbreviations

AE: adverse event; COX: cyclooxygenase; CSULGIEs: clinically significant upper and lower GI events; GI: gastrointestinal; ns: nonselective; NSAID: nonsteroidal antiinflammatory drug; OA: osteoarthritis; RA: rheumatoid arthritis; RCT: randomized controlled trial.

## Competing interests

AM has received research grants, consulting or lecture fees from pharmaceutical companies, including AstraZeneca, Eli Lilly, Flynn Pharma, GlaxoSmithKline, Grünenthal, Horizon, Menarini, MSD, Pfizer, and Reckitt Benkiser, for work involving analgesic drugs, but received no remuneration for work on this manuscript. GM and CL are full-time employees of Pfizer Inc and own stock and shares in Pfizer Inc. The manuscript was funded by Pfizer Inc.

## Authors' contributions

AM contributed to the conception and design, analysis, and interpretation of the data. GM contributed to the conception and design and interpretation of the data. CL contributed to the analysis and interpretation of the data. All authors contributed to the drafting of the manuscript and its revision for critically important intellectual content, and gave final approval of the version to be published.

## Supplementary Material

Additional file 1 **Clinical studies included in the pooled analysis**. A list containing the clinical studies included in the pooled analysis and the duration of treatment and treatment groups of each clinical study. Click here for file

Additional file 2 **Kaplan-Meier plot of time to incidence of CSULGIEs or symptomatic ulcers**. The time to incidence of CSULGIEs was significantly longer with celecoxib than with nsNSAIDs. CSULGIEs, clinically significant upper and lower GI events; nsNSAID, nonselective nonsteroidal antiinflammatory drug. Click here for file

Additional file 3 **Kaplan-Meier plot of time to incidence of CSULGIEs, symptomatic ulcers, or hemoglobin decrease ≥ 2 g/dl of GI-related or potential GI cause**. The time to incidence of CSULGIEs was significantly longer with celecoxib than with nsNSAIDs. CSULGIEs, clinically significant upper- and lower-GI events; GI, gastrointestinal; nsNSAID, nonselective nonsteroidal antiinflammatory drug. Click here for file
