# Supplementary material for: Patient-level pooled analysis of adjudicated gastrointestinal outcomes in celecoxib clinical trials: meta-analysis of 51,000 patients enrolled in 52 randomized trials
Source: Arthritis Res Ther. 2013 Jan 8;15(1):R6. doi: 10.1186/ar4134 (PMC3672676; doi:10.1186/ar4134)
Supplement: Additional file 1 — Clinical studies included in the pooled analysis. A list containing the clinical studies included in the pooled analysis and the duration of treatment and treatment groups of each clinical study. [file ar4134-S1.DOCX]

**Additional file 1** Clinical studies included in the pooled analysis

|  | Duration of treatment | Treatment groups |
| --- | --- | --- |
| **Osteoarthritis and/or rheumatoid arthritis** |  |  |
| A3191006 | 52 weeks | Celecoxib 200 mg TDD, diclofenac 50 mg BID |
| A3191025 | 1 year^a^ | Celecoxib 200 mg TDD, diclofenac 50 mg BID |
| A3191051 | 6 weeks | Placebo, celecoxib 200 mg TDD, naproxen 500 mg BID |
| A3191052 | 6 weeks | Placebo, celecoxib 200 mg TDD, naproxen 500 mg BID |
| A3191053 | 6 weeks | Placebo, celecoxib 200 mg TDD, naproxen 500 mg BID |
| A3191062 | 6 weeks | Placebo, celecoxib 200 mg TDD, ibuprofen 800 mg TID |
| A3191063 | 6 weeks | Placebo, celecoxib 200 mg TDD, ibuprofen 800 mg TID |
| A3191069 | 6 weeks | Placebo, celecoxib 200 mg TDD |
| A3191082 | 6 weeks | Placebo, celecoxib 200 mg TDD |
| A3191152 | 6 months | Celecoxib 200 mg TDD, naproxen 500 mg BID |
| I49-96-02-041 | 24 weeks | Celecoxib 400 mg TDD, diclofenac SR 75 mg BID |
| I49-96-02-042 | 6 weeks | Celecoxib 200 mg TDD, celecoxib 400 mg TDD, diclofenac 50 mg BID |
| I49-98-02-096 | 12 weeks | Celecoxib 200 mg TDD, celecoxib 400 mg TDD, diclofenac 50 mg BID, naproxen 500 mg BID |
| I49-98-02-105 | 12 weeks | Celecoxib 200 mg TDD, diclofenac 50 mg BID |
| I49-98-02-106 | 12 weeks | Celecoxib 200 mg TDD, diclofenac 50 mg BID |
| I49-98-02-107 | 12 weeks | Celecoxib 200 mg TDD, diclofenac 50 mg BID |
| J49-01-02-216 | 4 weeks | Placebo, celecoxib 200 mg TDD, loxoprofen 60 mg TID |
| N49-00-02-181 | 6 weeks | Celecoxib 200 mg TDD, rofecoxib 25 mg QD |
| N49-96-02-012 | 4 weeks | Placebo, celecoxib 400 mg TDD, celecoxib 800 mg TDD, celecoxib <200 mg TDD |
| N49-96-02-020 | 12 weeks | Placebo, celecoxib 200 mg TDD, celecoxib 400 mg TDD, celecoxib <200 mg TDD, naproxen 500 mg BID |
| N49-96-02-021 | 12 weeks | Placebo, celecoxib 200 mg TDD, celecoxib 400 mg TDD, celecoxib <200 mg TDD, naproxen 500 mg BID |
| N49-96-02-022 | 12 weeks | Placebo, celecoxib 200 mg TDD, celecoxib 400 mg TDD, celecoxib 800 mg TDD, naproxen 500 mg BID |
| N49-96-02-023 | 12 weeks | Placebo, celecoxib 200 mg TDD, celecoxib 400 mg TDD, celecoxib 800 mg TDD, naproxen 500 mg BID |
| N49-96-02-047 | 4 weeks | Placebo, Celecoxib 200 mg TDD, Celecoxib 400 mg TDD, Celecoxib 800 mg TDD |
| N49-96-02-054 | 12 weeks | Placebo, celecoxib 200 mg TDD, celecoxib 400 mg TDD, celecoxib <200 mg TDD, naproxen 500 mg BID |
| N49-96-02-060 | 6 weeks | Placebo, celecoxib 200 mg TDD |
| N49-97-02-062 | 12 weeks | Celecoxib 400 mg TDD, naproxen 500 mg BID |
| N49-97-02-071 | 12 weeks | Celecoxib 400 mg TDD, diclofenac 75 mg BID, ibuprofen 800 mg TID |
| N49-98-02-035 | 15 months | Celecoxib 800 mg TDD, ibuprofen 800 mg TID, diclofenac 75 BID |
| N49-98-02-087 | 6 weeks | Placebo, celecoxib 200 mg TDD |
| N49-98-02-102 | 15 months | Celecoxib 800 mg TDD, ibuprofen 800 mg TID, diclofenac 75 BID |
| N49-98-02-118 | 6 weeks | Placebo, celecoxib 200 mg TDD, diclofenac 50 mg TID |
| N49-99-02-149 | 6 weeks | Celecoxib 200 mg TDD, rofecoxib 25 mg QD |
| N49-99-02-152 | 6 weeks | Placebo, celecoxib 200 mg TDD, rofecoxib 25 mg QD |
| 635-IFL-0508-002 | 12 weeks | Celecoxib 200 mg TDD, rofecoxib 25 mg QD, naproxen 500 mg BID |
| 635-IFL-0508-003 | 6 weeks | Placebo, celecoxib, 200 mg TDD, rofecoxib 25 mg QD |
| COXA-0508-261 | 12 weeks | Celecoxib, 200 mg TDD, diclofenac 50 mg TID |
| **Ankylosing spondylitis** |  |  |
| COXA-0508-243 | 12 weeks | Celecoxib 200 mg TDD, celecoxib 400 mg TDD, diclofenac 75 mg SR BID |
| COXA-0508-247 |  | Celecoxib 200 mg TDD, celecoxib 400 mg TDD, diclofenac 50 mg TID |
| F49-98-02-137 | 6 weeks | Placebo, celecoxib 200 mg TDD, ketoprofen 100 mg BID |
| N49-01-02-193 | 12 weeks | Placebo, celecoxib 200 mg TDD, celecoxib 400 mg TDD, naproxen 500 mg BID |
| **Chronic low back pain** |  |  |
| A3191174 | 4 weeks | Celecoxib 400 mg TDD, loxoprofen 60 mg TID |
| COXA-0508-244 | 12 weeks | Placebo, celecoxib 200 mg TDD |
| COXA-0508-245 | 12 weeks | Placebo, celecoxib 200 mg TDD |
| COXA-0508-269 | 12 weeks | Placebo, celecoxib 200 mg TDD, celecoxib 400 mg TDD |
| J49-01-02-217 | 4 weeks | Celecoxib 200 mg TDD, loxoprofen 60 mg TID |
| **Alzheimer’s disease** |  |  |
| IQ5-97-02-001 | 52 weeks | Placebo, celecoxib 400 mg TDD |
| EQ5-98-02-002 | 3 years^a^ | Placebo, celecoxib 400 mg TDD |
| NQ5-98-02-005 | 4 weeks | Placebo, celecoxib 400 mg TDD, celecoxib 800 mg TDD, celecoxib <200 mg BID |
| **Cancer** |  |  |
| EQ4-00-02-018 | 3 years^a^ | Placebo, celecoxib 400 mg TDD |
| IQ4-99-02-005 | 3years^a^ | Placebo, celecoxib 400 mg TDD, Celecoxib 800 mg TDD |
| NQ4-00-02-011 | 12 weeks | Placebo, celecoxib 200 mg TDD, celecoxib 400 mg TDD, celecoxib 800 mg TDD |

^a^Treatment with study medication was suspended or terminated early.

BID, twice a day; QD, every day; TID, three times a day; TDD, total daily dose.
