# Supplementary figures and images for: Patient-level pooled analysis of adjudicated gastrointestinal outcomes in celecoxib clinical trials: meta-analysis of 51,000 patients enrolled in 52 randomized trials
Source: Arthritis Res Ther. 2013 Jan 8;15(1):R6. doi: 10.1186/ar4134 (PMC3672676; doi:10.1186/ar4134)

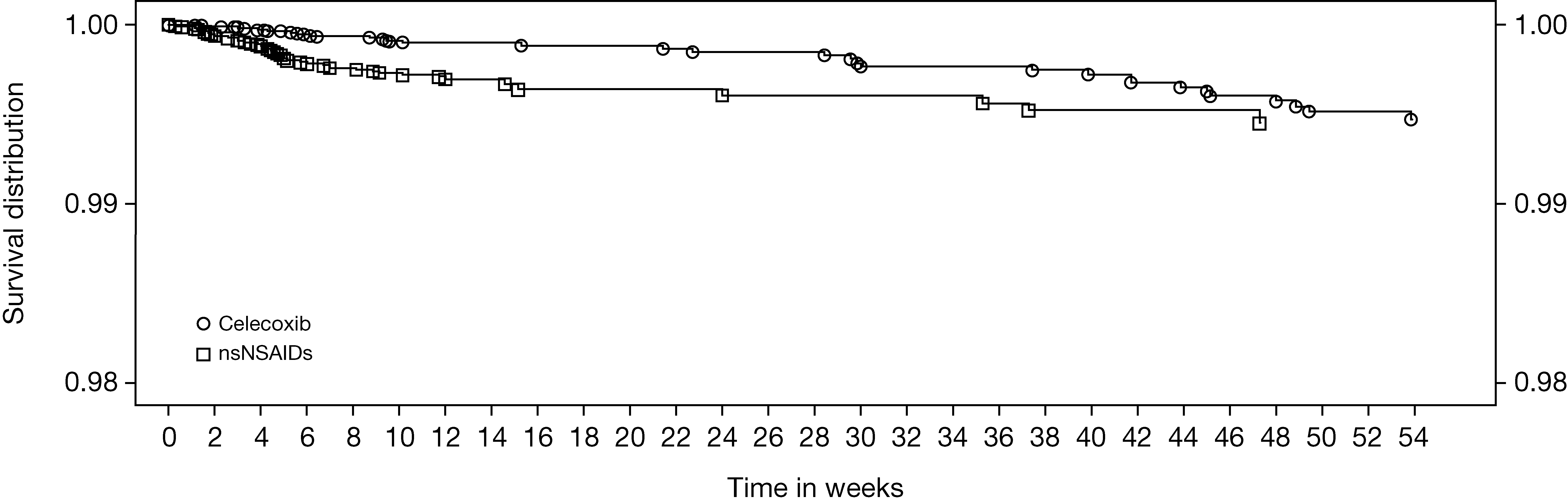

Supplement: Additional file 2 — Kaplan-Meier plot of time to incidence of CSULGIEs or symptomatic ulcers. The time to incidence of CSULGIEs was significantly longer with celecoxib than with nsNSAIDs. CSULGIEs, clinically significant upper and lower GI events; nsNSAID, nonselective nonsteroidal antiinflammatory drug. [file ar4134-S2.TIFF]

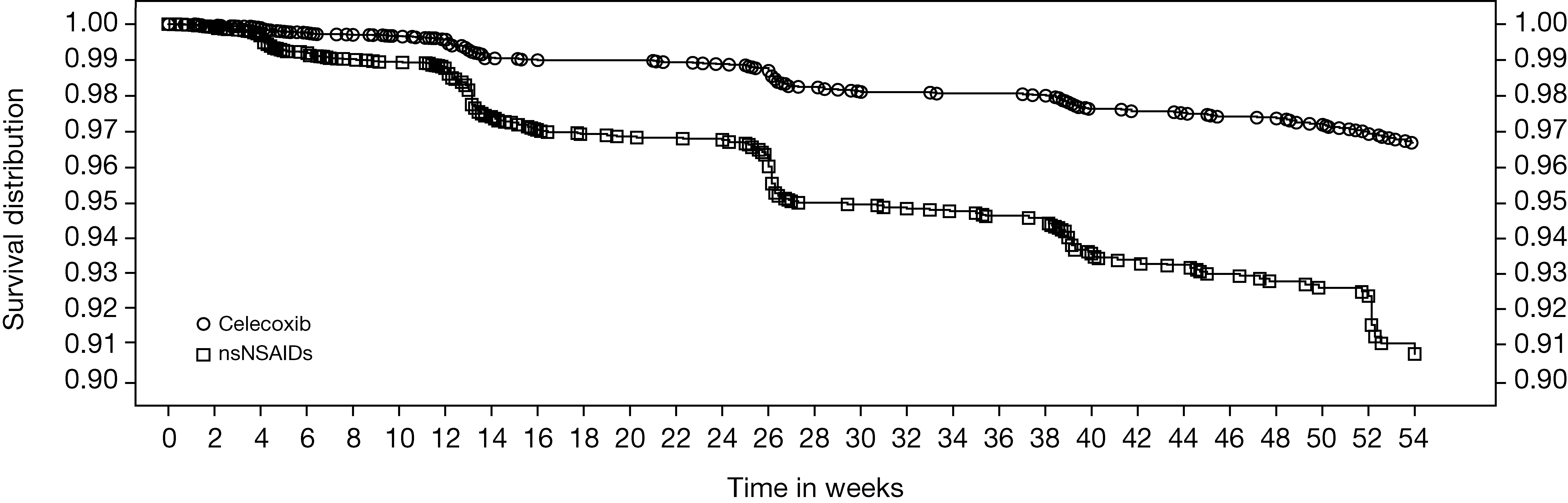

Supplement: Additional file 3 — Kaplan-Meier plot of time to incidence of CSULGIEs, symptomatic ulcers, or hemoglobin decrease ≥ 2 g/dl of GI-related or potential GI cause. The time to incidence of CSULGIEs was significantly longer with celecoxib than with nsNSAIDs. CSULGIEs, clinically significant upper- and lower-GI events; GI, gastrointestinal; nsNSAID, nonselective nonsteroidal antiinflammatory drug. [file ar4134-S3.TIFF]
